# Candidate gene identification of ovulation-inducing genes by RNA sequencing with an *in vivo* assay in zebrafish

**DOI:** 10.1371/journal.pone.0196544

**Published:** 2018-05-01

**Authors:** Wanlada Klangnurak, Taketo Fukuyo, M. D. Rezanujjaman, Masahide Seki, Sumio Sugano, Yutaka Suzuki, Toshinobu Tokumoto

**Affiliations:** 1 Integrated Bioscience Section, Graduate School of Science and Technology, National University Corporation Shizuoka University, Ohya 836, Suruga-ku, Shizuoka, Japan; 2 Department of Biological Science, Faculty of Science, National University Corporation Shizuoka University, Shizuoka, Japan; 3 Biological Science Course, Graduate School of Science and Technology, National University Corporation, Shizuoka University, Oya 836, Suruga-ku, Shizuoka, Japan; 4 Department of Computational Biology and Medical Sciences, Graduate School of Frontier Sciences, The University of Tokyo, Chiba, Japan; National Center for Toxicological Research, UNITED STATES

## Abstract

We previously reported the microarray-based selection of three ovulation-related genes in zebrafish. We used a different selection method in this study, RNA sequencing analysis. An additional eight up-regulated candidates were found as specifically up-regulated genes in ovulation-induced samples. Changes in gene expression were confirmed by qPCR analysis. Furthermore, up-regulation prior to ovulation during natural spawning was verified in samples from natural pairing. Gene knock-out zebrafish strains of one of the candidates, the starmaker gene (*stm*), were established by CRISPR genome editing techniques. Unexpectedly, homozygous mutants were fertile and could spawn eggs. However, a high percentage of unfertilized eggs and abnormal embryos were produced from these homozygous females. The results suggest that the *stm* gene is necessary for fertilization. In this study, we selected additional ovulation-inducing candidate genes, and a novel function of the *stm* gene was investigated.

## Introduction

Ovulation is a critical biological process that prepares fertilizable eggs prior to spawning. During this phase matured oocytes rupturing from the follicle layer require multiple factors, which regulate the phenomenon [1–5]. Vasotocin is prerequisite for the resumption of meiosis and ovulation in trout and catfish [3, 6–8]. The binding of prostaglandin E2 (PGE_2_) on the prostaglandin E2 receptor 4 (EP4) is required for ovulation in medaka [1, 9]. Ovulation is mediated by the nuclear progesterone receptor (nPR) through genomic steroid signaling mechanisms in zebrafish [4, 5]. A number of ovulation-associated genes involved in the luteinizing hormone (LH) signaling pathway have also been reported [4].

Ovulation-associated genes are expressed with maturation-associated genes in the ovary. The examination of only ovulation-associated genes in ovarian tissue has not been possible. This obstacle severely limits the study of ovulation. However, we previously established a way to select only ovulation-associated genes by using separate *in vivo* oocyte maturation and ovulation inductions [10]. We established a procedure that enabled the preparation of ovarian tissue containing oocyte maturation-induced oocytes in vivo by treating fish with diethylstilbestrol (DES) or testosterone (Tes). Similarly, ovulation can be induced in live zebrafish by treatment with 17α, 20β-dihydroxy-4-pregnen-3-one (17, 20β-DHP) [11]. This technique makes it possible to select the up-regulated genes that induce ovulation by comparing the gene expression between genes associated with oocyte maturation in ma- tured oocytes and both genes associated with oocyte maturation and ovulation in ovulated eggs. We proposed three ovulation-related candidate genes using microarray analysis in that study. Highly up-regulated genes at the time of ovulation were seen to be associated with apoptosis, and the results support the notion that apoptosis is a potential mechanism for inducing ovulation.

Recently, we began exploring other technologies to analyze gene expression profiling. Although, microarray analysis has been the traditional approach to analyze gene expression profiles over the last few decades, mRNA cross hybridization has been a major concern. We selected candidate ovulation-inducing genes using microarrays in a previous study [11]. However, the expression levels of most of the genes were inconsistent when checked by qPCR. To select ovulation-associated genes specifically, it is necessary to apply a more reliable technique. A new method for gene selection, RNA sequencing (RNA-seq), has been established to reveal the precise cellular transcriptome at any specific time using sequencing-based methods. RNA-seq is expected to provide a more reliable candidate screen for ovulation-related genes.

In this study, gene expression levels were compared among an ethanol (EtOH)-treated group (non-activated group), a DES- or a Tes-treated group (maturation-induced group), and a 17, 20β-DHP-treated group (maturation- and ovulation-induced group) by RNA-seq. Eight genes showing ovulation-specific up-regulation were identified. Among these, genome editing using the CRISPR/Cas9 system was performed on the starmaker (*stm*) gene [12]. We also investigated a novel function of *stm* gene in fertilization using mutant phenotype analysis.

## Materials and methods

### Animals

Zebrafish used in this study were the roy orbison (*roy*) strain. The strain is highly transparent, and the oocytes are easily observed in living fish. The characteristic makes it possible to select fishes ready for spawning. All fish were bred in our laboratory. Fish husbandry procedures were conducted following standard procedures [13]. Larvae were fed *Paramecium* spp. Over one month-old-fish were fed live brine shrimp in the morning and Tetra Guppy, flake fish food, (Tetra GmbH, Melle, Germany) in the evening. Fish were kept in a recirculating water system at 28.5°C under a controlled photoperiod (14 hours light: 10 hours dark cycle).

### Ethics statement

All zebrafish experiments were carried out with approval from the Institutional Ethics Committee of Shizuoka University, Japan (approved No. 29F-2).

### Collecting ovarian samples

#### Artificial induction of maturation and ovulation *in vivo*

To compare the gene expression profiles in ovaries, four kinds of ovarian samples (EtOH, DES, Tes, and 17, 20β-DHP -treated) were prepared by *in vivo* assay as described previously [10, 11]. More than four fish were used for each treatment. Female zebrafish were treated for three hours. After incubation, the female zebrafish were killed by spinal severance followed by dissection. The whole ovary from one side of each treated, sacrificed female was taken. Ovarian samples were frozen immediately with liquid nitrogen. The quality of the ovarian samples was checked by observation of the other side’s ovary before RNA extraction under a stereomicroscope. Oocyte maturation was assessed by scoring the oocytes that became transparent. Ovulation was assessed by scoring oocytes that showed a clear fertilization membrane. The specimen showing the best ovarian status based on observation after collection in each treatment (100% of immature oocytes for EtOH treatment, more than 90% of matured oocytes for DES or Tes treatment, more than 90% of ovulated eggs for 17,20β-DHP treatment) was selected for RNA sample preparation. Three replicates were separately prepared from three different batches (more than 20 individuals each) of zebrafish. Morphological characteristic of oocytes and eggs, after EtOH, DES, Tes, and 17,20β-DHP treatment were described previously [10].

#### Natural induction of maturation and ovulation

A male and a female were crossed in a plastic aquarium paved with glass beads or in a bottom-meshed aquarium from the evening until the morning of the next day. Ovaries were collected at 5 and 8 am. As described above, we maintain zebrafish under a 14-hour light condition. We set lights to turn off at 11 pm and turn on at 9 am. If pairings are successful, ovarian samples from 5 am are full of matured oocytes, and from 8 am are full of ovulated eggs. Three bio-replicates were collected.

### RNA extraction

Total RNA was extracted from one side’s ovary using ISOGEN (Nippon Gene, Tokyo, Japan) in accordance with the manufacturer’s protocol.

### RNA sequencing

The RNAs from fishes after four kinds of treatment (EtOH, DES, Tes, and 17, 20β-DHP) were used for Illumina RNA-seq analysis. RNA fractions were purified using RNEasy® mini kit (Qiagen, Valencia, CA). The cDNA of each sample was prepared with TruSeq RNA-seq kits (Illumina, Inc., Tokyo, Japan) from 1 μg of RNA according to the manufacturer’s instructions. Using the Illumina GAIIx, all libraries were sequenced on a single lane of 36-bp single-end sequencing according to the manufacturer’s protocol (Illumina, Inc., Tokyo, Japan). To count the number of transcripts, RNA-seq reads were aligned to the zebrafish genome sequence (danRer7) using Illumina ELAND v2 mapping software [14] (Table 1).

**Table 1:** Summary of RNA-seq.

Biological replicate	sample name	# Reads	uniq map (seed 32, mismatch2)	% of uniq map	No. of represented genes (Total)	No. of represented genes (RPKM > 0)	No. of represented genes (RPKM > 1)
1	EtOH_1	21,986,129	17,371,766	79%	15865	13,372	12,407
	DES_1	31,568,904	24,827,164	79%	15865	13,624	9,205
	Tes_1	19,925,529	15,951,889	80%	15865	13,259	9,240
	17, 20β-DHP_1	25,196,538	20,090,159	80%	15865	13,637	9,351
2	EtOH_2	32,000,909	24,959,619	78%	15865	13,755	9,401
	DES_2	42,557,042	33,294,822	78%	15865	13,581	9,025
	Tes_2	38,989,411	30,741,391	79%	15865	13,839	9,386
	17, 20β-DHP_2	42,422,071	33,424,477	79%	15865	13,912	9,328
3	EtOH_3	40,466,435	30,016,408	74%	15865	13,889	9,308
	DES_3	22,925,036	17,487,847	76%	15865	13,320	9,117
	Tes_3	38,410,087	29,143,517	76%	15865	13,761	9,178
	17, 20β-DHP_3	26,935,561	20,626,033	77%	15865	13,747	9,412
average	EtOH	31,484,491	24,115,931	77%	15865	13,672	10,372
	DES	32,350,327	25,203,278	78%	15865	13,508	9,116
	Tes	32,441,676	25,278,932	78%	15865	13,620	9,268
	17, 20β-DHP	31,518,057	24,713,556	78%	15865	13,765	9,364

### Gene selection

**Fig 1:**
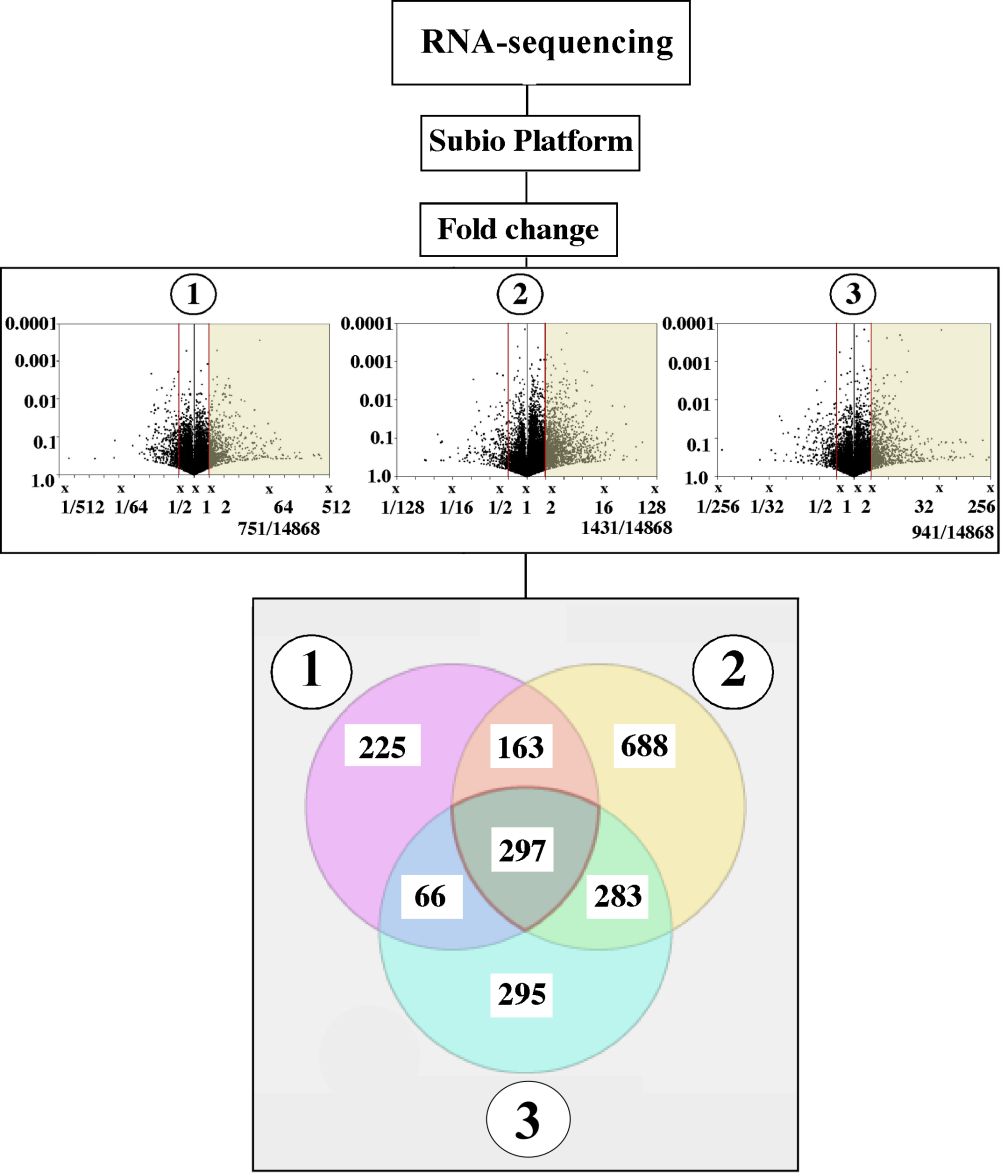
The process for selecting candidate genes associated with ovulation. Genes showing more than 2-fold greater expressions in 17, 20β-DHP than in EtOH (1), DES (2), or Tes (3) were selected. Overlapping genes among these three groups were selected by Venn diagrams analysis.

The mean values of gene expression levels in the samples treated with EtOH, DES, Tes, and 17, 20β-DHP from the RNA sequencing were analyzed by Subio Platform ver. 1.18.4667 (Subio Inc., Amami, Japan). Subio Platform is free software supporting biologists’ interpreting, discussing on and sharing microarray or NGS data. Researchers can add generally-used analysis tools by purchasing Plug-ins, and work with R and Bioconductor to apply more complicated analyses. Subio Platform is designed to visualize data itself at multi-angles and–layers. That make it possible to examine how the process affects the data at each step on desk top personal computer. A non-statistical selection method was used to select genes up-regulated in 17, 20β-DHP treated samples [10]. That is, any genes that showed two-fold higher expression in the 17, 20β-DHP treated group when compared with the other three treatment groups (EtOH, DES, and Tes) were identified as ovulation-inducing gene candidates (Fig 1).

### qPCR

Expression levels of candidate genes were confirmed by qPCR. Total RNA (1 μg) was reverse transcribed using Illustra Ready-To-Go RT-PCR Beads (GE Healthcare Life Sciences, Buckinghamshire, UK) according to the manufacturer’s instructions. The qPCR reaction mixtures (20 μl) included 1 μl of each primer (10 μM), 10 μl of SYBR green PCR Master Mix (Roche Applied Science, Mannheim, Germany), and 5 μl of cDNA (10-fold diluted). Real-time qPCR was conducted by LightCycler® Nano System (Roche Applied Science, Mannheim, Germany). The thermal cycle was as follows: 95°C for 5 minutes, 45 cycles of 95°C for 10 seconds, annealing temperature (Ta) (Table 2) for 10 seconds, and 72°C for 15 seconds. The final melting curve analysis was performed at 65°C for 20 seconds, followed by 95°C for 20 seconds. Triplicates were done for each individual sample. The mRNA expression level of each target gene was normalized against elongation factor 1α (EF1α) expression level (a common reference gene). The normalized mRNA abundances were reported as the mean ± SE. The normalized mRNA abundances were reported as the mean ± SE. Specific primers were designed via Primer 3 [15] (Table 2). All of primers used for qPCR analysis were designed to span exon-exon junction.

**Table 2:** Primers used for qPCR analyses of eight candidates and one positive gene in the RNA-seq selection.

Target gene	Accession #	Forward Primer 5' to 3'	Reverse Primer 5' to 3'	product size (bp)	Ta (°C)
*ctrb1*	NM_212618	CAATGAGAATTGGGTTGTGACTGC	TGTTGTAGTTAGGATGCTTGATGGA	146	55
*prss59*.*1*	NM_199605	CTGCTCACTGCTACAAGTCAC	GTCAAGATCCCACGAGTCATAG	134	56
*ctsbb*	NM_001110478	GATGAATGTGGGATTGAGAGTGAGA	ATTGTGTTTGAGCGTGATTTAGAGG	120	58
*stm*	NM_198817	ATCTCTGATGTCTTTGTCTATGTGG	CAGTCTTTATTAGATTTGCTCCCTG	149	60
*adamts15a*	NM_001126429	GAGAGCAAAGATAACAAGGCACAAA	TTTTCCACCTTTATTGACTCCACCT	96	56
*sik1*	NM_001126383	AGAGAAGACCAAAGTTGATT	ATAGGATGGCTAGTATTGTG	154	60
*pax2a*	NM_131184	TAATGCTTGCGGTCCCTTAAATATG	ATCAGTCCATTCAACGAAGACACG	149	61
*rbm47*	NM_001114686	CCAGAGAGGCACAATCTTAATGTCA	GACTACAGCACAGAGACGAGTTAAA	109	57
*ptgs2a*	NM_153657	ATGTTTGCTTTCTTCGCCCA	AGATCCACTCCATGACCCAG	101	55

### Genome editing by CRISPR/Cas9 system

Template oligonucleotides for single guide RNA (sgRNA) synthesis (Invitrogen^TM^ GeneArt^TM^ Strings^TM^ DNA fragments) were obtained from Thermo Fisher Scientific Inc. (Waltham, MA, USA). The sgRNA for the stm gene was synthesized *in vitro* from template oligonucleotide according to the manufacturer’s instructions using the MEGA shortscript^TM^ Kit (Thermo Fisher Scientific Inc., Waltham, MA, USA). Cas9 protein was obtained from Clontech Laboratories (Takara Bio, Japan).

A mixture of the *stm* sgRNA (0.54 μg/μl) and Cas9 nuclease (500 ng/μl) was injected into the one cell stage embryo of zebrafish in 4 nl of volume. The injected embryos (F0) were cultured in petri dishes at 28.5°C for 5 days. The fry were released into a culturing tank after they started to swim continuously. The embryos were raised to adulthood.

### Genotyping by Heteroduplex mobility assay (HMA) and sequencing

Heteroduplex mobility assay (HMA) was carried out to evaluate the genotype of mutants [16]. Genomic DNA was extracted from a portion of the caudal fin by phenol-chloroform extraction. Primers were designed using Primer 3 software [15]. A 198 bp amplicon, which covers the target site, was amplified. A 25 μl PCR reaction mixture included 0.1 μl (0.5 U) of Ex *Taq* DNA Polymerase, 2.5 μl of 10X Ex *Taq* Buffer (Mg^2+^ -Plus), 2 μl of 2.5 mM dNTP mix (Clontech Laboratories, Takara bio, USA), 2.5 μl of 10 mM forward (5′-CACACACAACGAGCAGACGTGAAGA-3′) and reverse primer (5′-TGTGTGTGTGTGTGCGCTTGTGAAA-3′), and 4 μl of genomic DNA template. Three-step PCR was performed: 95°C for 5 minutes, 35 cycles of 95°C for 30 seconds, 58°C for 30 seconds, and 72°C for 1 minute. 5 μl of PCR product was mixed with 3 μl of 10X loading buffer. Mixed samples were heated at 95°C for 5 minutes and immediately put into wet ice. Electrophoresis was done on 15% non-denaturing polyacrylamide gels at 150 V, 30 mA, for more than two hours. The gel was stained with ethidium bromide for visualization.

To further analyze the mutant DNA sequences a 522 bp amplicon, which covers the target site, was amplified. Reaction mixtures (25 μl) contained 0.5 μl (0.5 U) of KOD (DNA polymerase from hyperthermophilic archaeon *Pyrococcus kodakaraensis*)-Plus, 2.5 μl of 10X Buffer for KOD -Plus, 2 μl of 2 mM dNTP mix, 0.5 μl of 25 mM MgSO4 (TOYOBO CO., LTD., Osaka, Japan), 2.5 μl of 10 μM forward (5′- GACGTACAAGTGGAAGTAACTCTGG-3′) and reverse primer (5′- TGCTGTAACTCCTGTAATCTTTTCC -3′), and 4 μl of genomic DNA template. Three-step PCR was performed: 95°C for 5 minutes, 35 cycles of 95°C for 30 seconds, 58°C for 30 seconds, and 72°C for 1 minute. Before sequencing, the reaction mixture was cleaned up using an ExoProStar PCR and Sequence Reaction Clean-Up Kit (GE Healthcare Life Sciences, Tokyo, Japan) according to the manufacturer’s instructions.

## Results

Illumina-based RNA sequencing (RNA-seq) was conducted on ovarian total RNA from EtOH, DES, Tes, or 17,20β-DHP -treated fish. Sequencing for each treatment was conducted in triplicate. Number of RNA-seq reads varied between 19 to 42 million reads (Table 1). Over 75% of reads mapped onto the reference genome. Among 15,865 annotated genes, around 13,000 genes were detected, and around 10,000 genes were reported as analyzable. Raw read data were submitted to the DNA data bank of Japan (DDBJ), deposited as Bio project no. PRJDB5490 (PSUB006856). Relative expression levels were calculated from the number of reads. Tag counts were normalized to read per million tags per kilobase mRNA (rpkm) and were used as the gene expression information.

### Ovulation-related genes selected from RNA sequencing platform

Genes that were up-regulated more than two-fold in the 17, 20β-DHP-treated group against EtOH- (1), DES- (2) and Tes- (3) treated groups, were selected (751, 1,431, and 941 genes, respectively) (Fig 1). Among these genes, significant up-regulation only occurred in the 17, 20β-DHP-treated group, which were selected by Venn Diagram analysis. 297 of these genes were reported S1 Table. One of the positive control, ovulation-related genes, prostaglandin-endoperoxide synthase 2a (*ptgs2a*), was found in this selection. Among these genes, we checked changes in expression levels for eight of the genes that presented a significant elevation in the 17, 20β-DHP-treated group (Table 3).

**Table 3:** Eight candidates for genes associated with ovulation selected by RNA-seq platform.

Gene name	Accession#	Fold change relative to EtOH			Fold change relative to DES			Fold change relative to Tes			Fold change relative to 17, 20β-DHP		
		DES	Tes	17, 20β-DHP	EtOH	TES	17, 20β-DHP	EtOH	DES	17, 20β-DHP	EtOH	DES	TES
*ctrb1* (chymotrypsin B1 precursor)	NM_212618	25.7	2.0	248.8	0.0	0.1	9.7	0.5	12.9	124.4	0.0	0.1	0.0
*prss59*.*1* (Danio rerio protease, serine, 59, tandem duplicate 2 (prss59.2), mRNA/trypsinogen precursor)	NM_199605	10.7	2.0	76.0	0.1	0.2	7.1	0.5	5.5	38.7	0.0	0.1	0.0
*csbb* (capthepsin B, b precursor)	NM_001110478	2.9	0.3	53.6	0.4	0.1	18.8	3.5	10.0	187.7	0.0	0.1	0.0
*stm* (protein starmaker precursor)	NM_198817	0.6	1.5	9.4	1.7	2.6	16.3	0.7	0.4	6.4	0.1	0.1	0.2
*adamts15a* (A disintegrin and metalloproteinase with thrombospondin motifs 15 precursor)	NM_001126429	2.3	2.5	8.5	0.4	1.1	3.8	0.4	0.9	3.4	0.1	0.3	0.3
*sik1* (salt-inducible kinase1)	NM_001126383	0.9	1.6	6.9	1.1	1.8	7.7	0.6	0.6	4.3	0.1	0.1	0.2
*pax2a* (paired box protein Pax-2a)	NM_131184	0.6	0.8	4.6	1.6	1.3	7.6	1.2	0.7	5.7	0.2	0.1	0.2
*rbm47* (RNA-binding motif protein 47, transcript variant X3)	NM_001114686	0.5	0.9	2.1	2.0	1.9	4.3	1.1	0.5	2.3	0.5	0.2	0.4
*ptgs2a* (prostaglandin-endoperoxide synthase 2 precursor)	NM_153657	0.5	0.8	34.6	2.2	1.8	75.0	1.2	0.5	40.9	0.0	0.0	0.0

The fold changes among treated samples are shown.

### Confirmation of gene expression by qPCR

**Fig 2:**
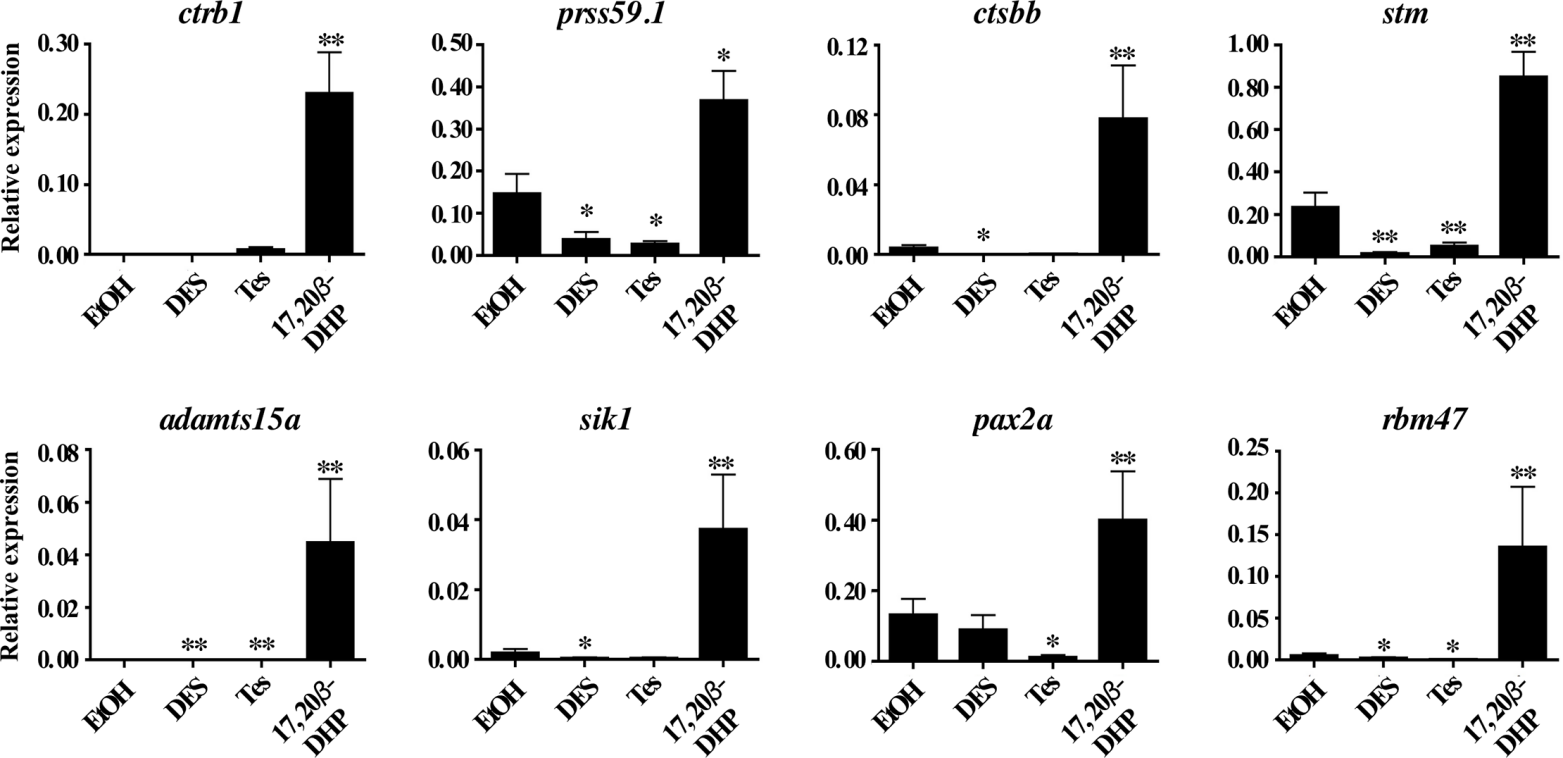
qPCR analysis of candidate genes associated with ovulation. Conducted using cDNAs prepared from ovaries of fish treated by EtOH, DES, Tes or 17, 20β-DHP for 3 hours. mRNA abundance was observed in triplicate for each sample, and all data were normalized by the number of elongation factor 1α (EF1α) transcripts in each sample. Expression values are represented as the mean ± SE of three independent samples. ** and * indicate statistically significant differences between the values for each treated group and EtOH-treated group at the P<0.01 and P<0.05 levels, respectively.

The expression levels of these eight 17, 20β-DHP-treated genes were analyzed by qPCR. All eight genes showed significantly greater expression in the 17, 20β-DHP-treated group (Fig 2, P < 0.05 with all other groups). Expression levels of the putative serine protease 59 tandem duplicate 1 (*prss59*.*1*), starmaker (*stm*), and paired box protein 2a (*pax2a*) were slightly elevated in ovaries from the EtOH-treated fish, however, these were still over two-fold lower than the 17, 20β DHP-treated fish.

### Further confirmation of gene expression by samples from natural pairings

**Fig 3:**
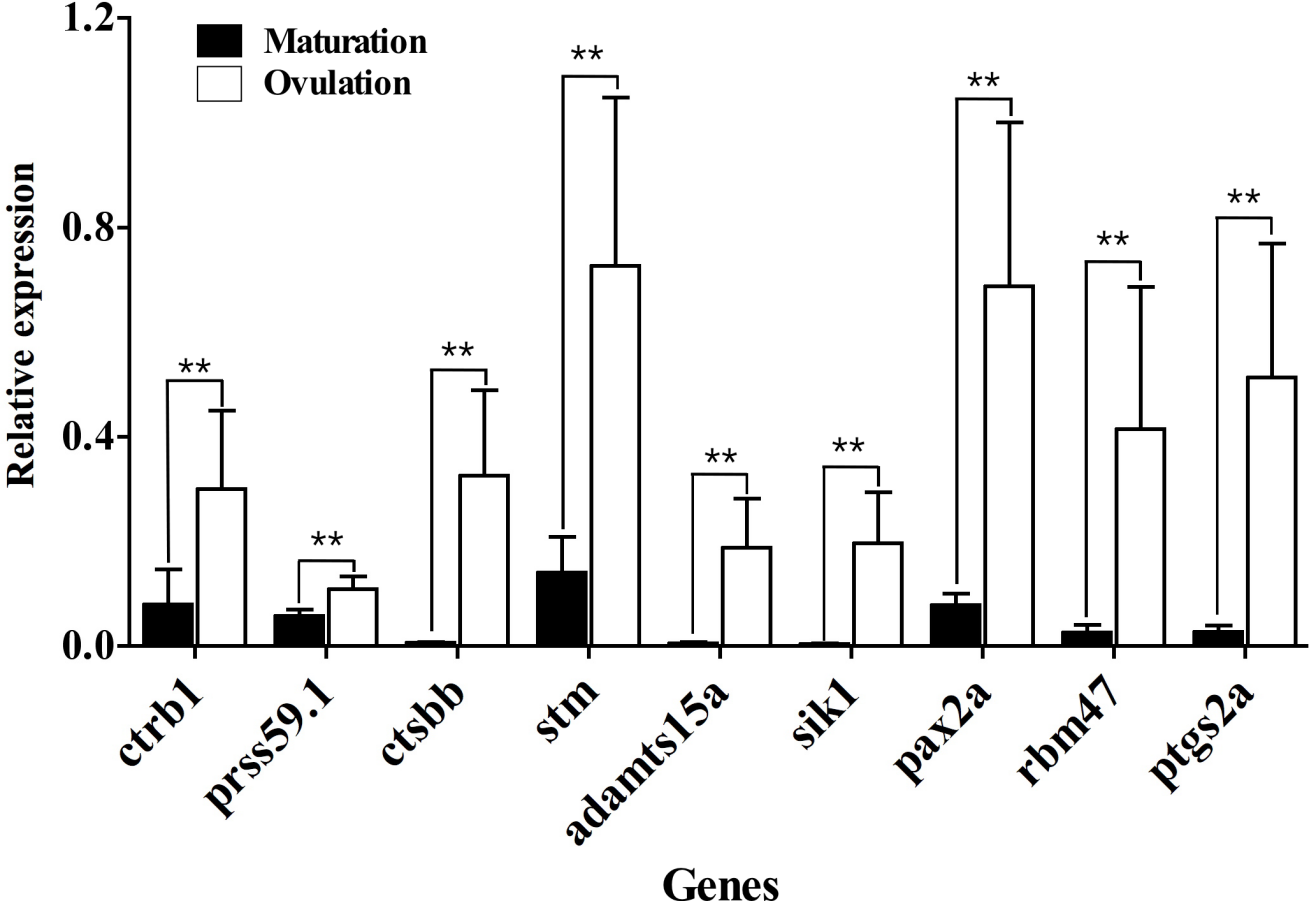
Changes in gene expression levels during natural paring. Relative expression of candidate genes in ovaries were compared between ovaries containing matured oocytes versus ovulated eggs sampled from females during natural pairings. mRNA abundance was observed in triplicate for each sample, and all data were normalized by the number of elongation factor 1α (EF1α) transcripts in each sample. Asterisks represent significant difference between the samples (** P ≤ 0.01).

To confirm the changes in expression levels of candidate genes during natural spawning, ovarian samples were prepared from females during natural pairing at 4 hours before (5 am) and 1 hour before (8 am) we turned on the light. The ovaries obtained 4 or 1 hour before lighting mainly contained matured oocytes or ovulated eggs, respectively. The elevation in mRNA abundance of nine genes (including the positive control gene, *ptgs2a*) was evaluated by comparing the expression in the ovary containing matured oocytes versus ovulated eggs (Fig 3). As expected, all genes showed higher expression in ovaries containing ovulated eggs, and were significantly different than that of the ovaries with matured oocytes. Expression of cathepsin Bb precursor (*ctsbb*), a disintegrin, and a metalloproteinase with a thrombospondin motif 15a precursor (*adamts15a*), salt-inducible kinase1 (*sik1*), RNA-binding motif protein 47 transcript variant X3 (*rbm47*), and *ptgs2a* were almost undetectable in ovaries with matured oocytes, but distinct expression was detected in the ovaries with ovulated eggs.

### Exploring the role of the *stm* gene

**Fig 4:**
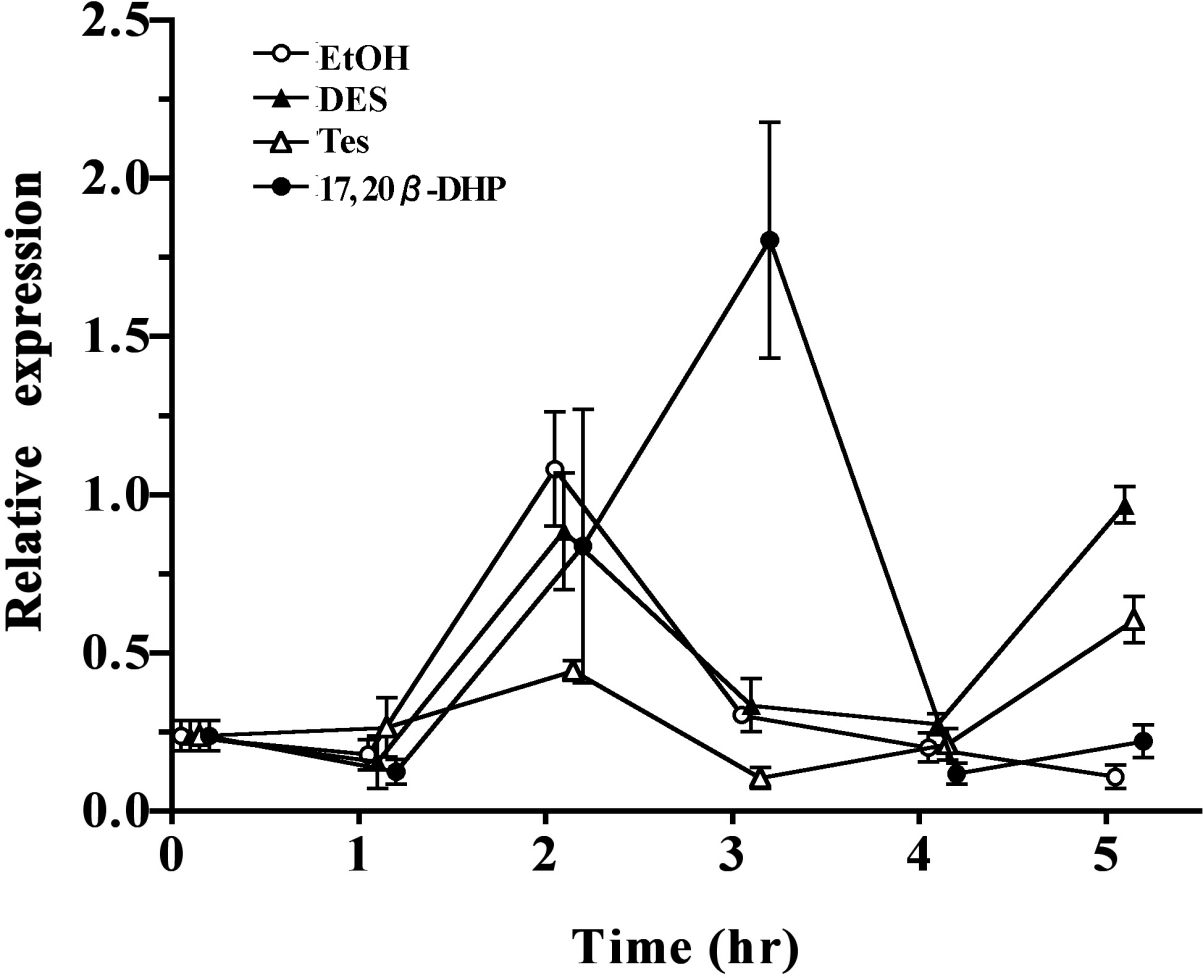
Time course changes of abundance of *stm* gene during in vivo treatments. Changes in the expression of *stm* mRNA for EtOH, DES, Tes, or 17, 20β-DHP-treated samples were analyzed in samples from 0 to 5 hours of treatment. All data were normalized by the number of elongation factor 1α (EF1α) transcripts in each sample. Expression values are represented as the mean ± SE for three independent samples.

As the first gene identified among the eight highly expressed 17, 20β-DHP-treated genes, we decided to analyze the role of *stm*. The *stm* gene expression profile in ovarian tissue during the time period from 0 to 5 hours with four kinds of treatment was investigated (Fig 4). At 2 hours of treatment, ovarian *stm* gene expression levels were primarily raised in the Tes and 17, 20β-DHP-treated fish. Expression levels in the 17, 20β-DHP-treated fish reached its peak at 3 hours. In contrast, expression levels in the Tes-treated fish went down to the basal level at 3 hours. The *stm* expression profile in the DES-treated fish did not fluctuate, and stayed at the same low level as the sample before treatment.

### Establishment of *stm* gene knock-out fish using the CRISPR/Cas9 system

**Fig 5:**
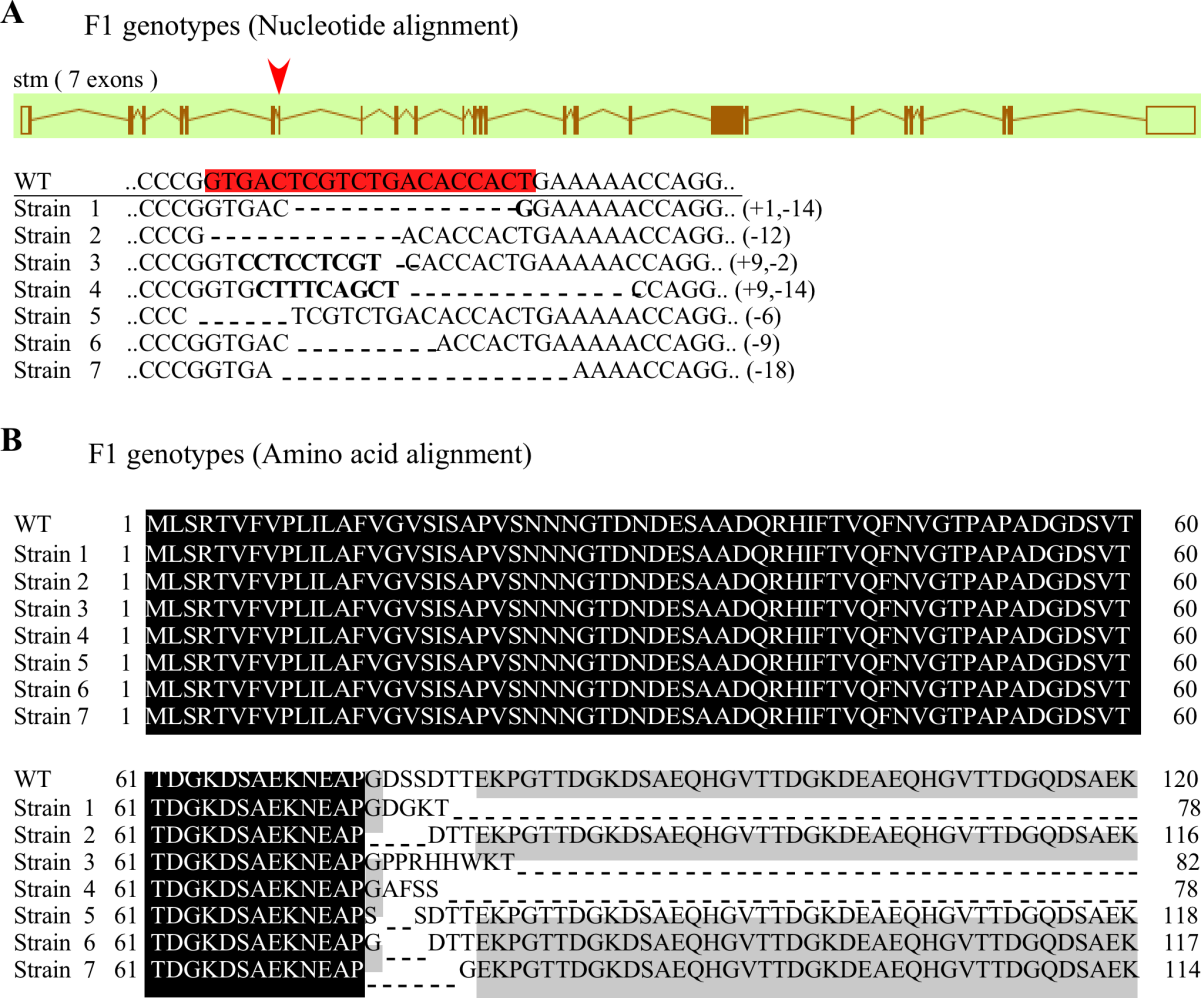
Genotype details of F1 progeny from *stm*-KO zebrafish. (A) The position of the target site is highlighted in red. Nucleotide alignment shows deletions (dashes) and insertions at the target site in the seven strains of heterozygous mutants. The numbers of deleted or inserted bases are indicated. (B) Amino acid alignment shows truncated peptides caused by frame shift mutations or deletions.

Gene expression profiles strongly suggest the role of the *stm* gene in the induction of ovulation. Thus, we proceeded to establish *stm* gene knock-out (KO) fish to show the physiological role of the *stm* gene. Mutant stains were generated by the injection of the CRISPR/Cas9 mixture into single cell stage embryos. The germline transmission efficiency of 11 germline-transmitting founders was 76%. Various mutations of the *stm* gene were found in F1 mutants, including in-frame deletions and/or frame shift mutations (Fig 5). This implies that we succeeded in generating *stm* knock-out fish using the CRISPR/Cas9 system. Among seven of the F1 mutant strains, we selected one strain for further investigation. This strain had one nucleotide insertion and 14 nucleotide deletions in the *stm* gene (strain 1 in Fig 5). Thirteen of the deletions resulted in a truncation of the STM protein (78 amino acids length compared to 613 amino acids in wild type) (Fig 5). Next *stm*^-/-^ mutants were generated by inbreeding between F1 heterozygotes (stm+/-) with the same *stm* mutation. HMA and sequence analysis suggest that 25% of the F2 progeny are *stm*^-/-^. This genotype ratio fits Mendelian rules, and results suggests that homozygous mutants are able to survive. External morphology and spawning behaviors of the *stm*^-/-^ mutants are no different from wild type (WT) (data not shown). Unexpectedly, *stm*-KO mutant (*stm*^-/-^) females are fertile, can ovulate eggs, and perform spawning.

### *stm*-KO mutant (*stm*^-/-^) females produce unfertilized eggs

**Fig 6:**
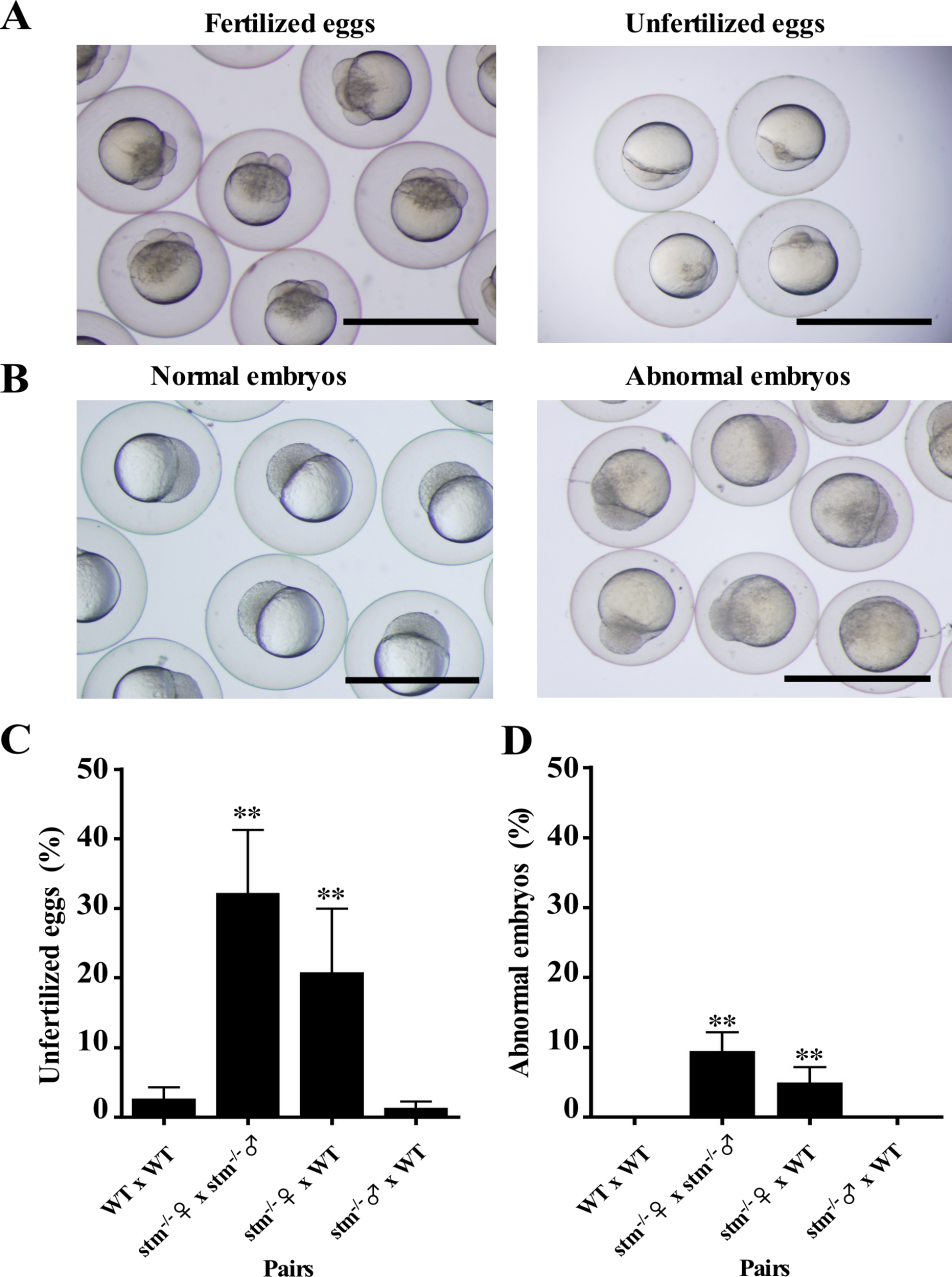
Morphology and rate of development of embryos from *stm* mutants. (A) Egg morphologies from *stm*^-/-^ female × *stm*^-/-^ male pairings were photographed. Fertilized (left) and unfertilized eggs (right) at 1.5 HPF were separated. Fertilized eggs reached four-cell stage, but unfertilized eggs remained in the first cell stage. The scale bars indicate 1 mm. (B) Normally developing embryos (left) and abnormal embryos (right) at 3 HPF were separated. The scale bars indicate 1 mm. (C) Percentage of unfertilized eggs in the eggs obtained from indicated four different sets of pairs. The fertilization rate (%) was calculated by determining the percentage of embryos that developed to four-cell or subsequent stages. Observations were done in triplicate. (D) Percentage of abnormal embryos among the embryos obtained from indicated four different sets of pairs. The percentage of abnormal embryos (%) was calculated by determining the percentage of embryos showing abnormal development among the fertilized eggs. **indicate statistically significant differences between the values for each paring and WT x WT paring at the P<0.01 level.

We observed the development of eggs to seek for egg deficiencies in the *stm*-KO mutants. Surprisingly, a high percentage of unfertilized eggs were seen with *stm*^-/-^ female × *stm*^-/-^ male pairs (32%), and with *stm*^-/-^ female × WT pairs (21%) (Fig 6). On the other hand, most of the eggs from wild-type pairs (WT × WT) and the *stm*^-/-^ male × WT pairs were fertilized (96% and 99%, respectively), and showed normal development. Furthermore, some of fertilized eggs from *stm*^-/-^ female × *stm*^-/-^ male pair (10%) and *stm*^-/-^ female × WT pair (5%) showed abnormal development at 3 hours post fertilization (HPF) (Fig 6B). In the case of the WT × WT pairs, most embryos were in transition between one-thousand-cell and later stages. Abnormal eggs are almost absent in the WT × WT pairs and the *stm*^-/-^ male × WT pairs (Fig 6D). Abnormal embryos showed an unusual morphology at 3 HPF. Most abnormal embryos showed ordinary cytoplasmic division at early cleavage stages. However, development was very slow, and some embryos stayed at 32-cell to 64-cell stages with irregular patterns of cell division. Some embryos developed into blastulas, but the blastodiscs showed distorted shapes, instead of the usual ball-like shape, and some parts of the blastodiscs did not properly develop (Fig 6B). Even though a higher percentage of unfertilized eggs and abnormal embryos appeared from the *stm* mutants, about half of the embryos continued normal development, hatched, and produced swimming juveniles. However, almost all *stm* mutant fish died during later cultivation. The results suggest that the *stm* gene possesses essential roles in fertilization and early development.

## Discussion

The *in vivo* bioassay established in our laboratory provides a novel way for distinguishing signaling pathways in the induction of ovulation [11]. Ovulation is induced by the genomic actions of 17, 20β-DHP concurrently oocyte maturation that is induced by the non-genomic actions of same steroid [11]. The preparation of ovarian samples with matured oocytes or ovulated eggs is a practical technique. Previously, we reported the selection of ovulation-related genes using this technique [10]. Ovarian sample gene expression profiles with matured oocytes versus ovulated eggs were compared by microarray analysis in that research. High levels of expression of maturation-related genes was detected in mRNAs extracted from matured oocytes. Likewise, both maturation-related and ovulation-related genes are highly expressed in ovarian samples with ovulated eggs. Therefore, genes that are highly expressed in ovulated eggs, but have low expression in matured oocytes are candidates for ovulation-inducing genes. Using microarray analysis we selected a number of genes up-regulated during ovulation [10]. However, many of those genes presented inconsistent expression levels between the microarray and qPCR analyses results. Cross hybridization of mRNA was suspected. This complication may lead to the misinterpretation of gene expression profiles [17]. Therefore, in the current study, RNA-seq, which is a modern approach for detecting transcriptome profiles, was adopted to provide a more biologically relevant and reliable gene expression profile. Eight genes (*ctrb1*, *prss59*.*1*, *ctsbb*, *stm*, *adamts15a*, *sik1*, *pax2a*, and *rbm47*), and one positive control gene (*ptgs2a*) were selected. All genes selected by RNA-seq show consistent up-regulation in qPCR analysis, both from the artificial (17, 20β-DHP-treated fish) and natural induction of ovulation. This guarantees that our candidate genes are specifically up-regulated at the beginning of ovulation.

Many biological processes, such as proteolysis, inflammation, coagulation, vasodilatation, and angiogenesis, have been suggested as essential mechanisms related to the acquisition of maturational competence and ovulation occurring in the pre-ovulatory ovarian follicle [18]. Several authors have tried to identify the genes responsible for ovulation. The first transcriptome analysis was conducted in rainbow trout (*Oncorhynchus mykiss*) [6]. Gene expression networks during ovarian development including ovulation were analyzed in largemouth bass (*Micropterus salmoides*) [19]. The gene sets that were up- or down-regulated were identified using microarray analysis. One of the genes selected in that study, *ctsbb*, which may relate to follicular atresia, was selected as inducing ovulation in largemouth bass. Specifically up-regulated genes in the pre-ovulatory, peri-ovulatory, and peri-spawning intervals have also been identified in zebrafish [20]. In our analysis, changes in the expression level of a positive control gene, *ptgs2a*, correlated well with this previous report. Very recently, Liu et al., reported differential gene expression analysis between wild type and nuclear progesterone receptor (nPR) gene knock-out zebrafish by RNA-seq analysis [21]. By comparing mRNA expression levels in follicle cells from these zebrafish, they identified genes up-regulated before ovulation that are thought to be up-regulated by the function of nPR. However, none of the genes they reported overlapped with the genes selected in our study. We believe that the genes we selected are mainly expressed in oocytes, because of our extraction of mRNAs from ovarian tissue. We attempted to catch a snapshot of RNA expression during ovulation by stopping changes in the amount of mRNAs in our samples by freezing the ovary tissue instantly using liquid nitrogen.

Genes selected in this study can be classified into three groups. The first group is composed of two genes (*ctsbb* and *sik1*), which can both trigger apoptosis. Apoptosis is one of the mechanisms involved in the induction of fish ovulation. Genes in the second group, the three candidates *zgc*:*65811*, *adamts15a*, and *rbm47*, may play roles in fertilization and embryogenesis. Four genes comprise the last group, and are newly identified players in the ovulation-inducing pathway (*pax2a*, *stm*, *ctrb1*, and *prss59*.*1*).

### Apoptosis: One of the mechanisms involved in induction of fish ovulation

Apoptosis has been shown to be an essential mechanism for follicle rupture, which leads to the completion of ovulation [22–24]. Two up-regulated genes found in this study may have roles in the induction of ovulation through the regulation of apoptosis.

Cathepsin B (*ctsbb*) promotes tumorigenesis and cancer progression in human cells [25–27]. It can mediate apoptosis by cleavage of proapoptotic proteins, such as BH3 interacting-domain death agonist (BID) [28], and it induces the degradation of antiapoptotic proteins (Bcl-2, Bcl-xL, or Mcl-1), which promote the mitochondrial pathway of apoptosis [29].

The gene for salt inducible kinase 1 (*Sik1*), is another that can mediate apoptosis [30, 31]. *Sik1* belongs to the adenosine monophosphate-activated kinase (AMPK) subfamily. AMPK proteins are implicated in cell metabolism [32]. AMPK proteins induce cytokinesis and cytotoxicity in CD8(+) T-cells toward islets in mice [33]. These mechanisms are involved in regulating β-cell apoptosis [33]. *Sik1* provokes P53 protein activity and subsequently induces anoikis, a subtype of apoptosis [30]. Resistance to apoptosis can be induced when cells lack SIK1 activity [30].

Apoptosis occurring prior to ovulation becomes an interesting mechanism in terms of regulating the rupture of an oocyte from its follicular layer [10]. Degradation of some follicular cells to create the rupture site prior to ovulation is potentially mediated by apoptosis. Thus, these two genes selected in our study may have roles in ovulation.

### Genes reported to have the role in embryogenesis

The *adamts15a* gene encodes a protease with a disintegrin-like and a metalloproteinase domain and thrombospondin-15 motifs. The zebrafish genome contains 17 *adamts* genes [34]. The mRNA of *adamts15a* is expressed ubiquitously in several organs, particularly heart, liver, and kidney. Expression levels of all *adamts* gene members were low in ovaries with oocytes. However, in comparison with other genes in this family, *adamts15a* is one of those genes highly up-regulated 24 hours post fertilization [34]. This gene plays a key role during embryogenesis. Biological functions of this gene during oogenesis remain unclear. Ours is the first evidence reporting high expression of *adamts15a* during fish ovulation.

Zebrafish RNA binding motif protein 47 (RBM47) is orthologous to human RBM47. RBM47 is ubiquitously expressed in many human tissues. Its gene plays an essential role during embryogenesis, including head formation and embryonic patterning [35]. We speculated this gene may have other functions prior to embryogenesis. While the up-regulation of *rbm47* has never been reported in the ovarian tissue of zebrafish, it has been described in the ovary of mature eel [36].

Therefore, we believe that *adamts15a* and *rbm47* are both up-regulated before fertilization to prepare for early development, and may not have roles in ovulation.

### New players in the ovulation-inducing pathway

Some genes up-regulated during ovulation that we identified in our study have never been directly reported to function in the reproductive system. This suggests the possibility of the existence of unidentified pathways in the induction of ovulation.

The Chymotrypsinogen B1 gene (*ctrb1*) encodes the principal precursor of a serine protease synthesized in the pancreas and continuously secreted to small intestine. Putative serine protease 59 tandem duplicate 1 (*prss59*.*1*) encodes trypsinogen 1a, a precursor for trypsin production in the pancreas. Proteases should play important roles in ovulation, as reported previously [37]. Further analysis is necessary to show the roles of these common digestive enzymes in the induction of ovulation.

The paired box gene (*pax2a*) encodes a transcription factor. It is expressed in the ovary of catfish, and has been speculated to be an up-stream regulator of the Wnt signaling pathway [38]. *Pax2a* also plays roles in maintaining the primordium and in the differentiation of thyroid follicles in zebrafish during early embryogenesis [39].

The starmaker gene (*stm*) plays an essential role in zebrafish otolith morphogenesis, regulating crystal growth and otolith shape [40, 41]. Starmaker is extremely expressed at the ear in early embryogenesis, and is subsequently expressed at the backbone in later stages [40]. The expression of this protein becomes a useful bio-indicator for the study of otolith formation in fish [41]. This is the first report of expression of *stm* in ovarian tissue. Consequently, *stm* function in the reproductive system is unknown. Interestingly, *pax2a* is an up-regulator of *stm*. A heat-inducible *pax2* construct was injected in medaka embryos to reveal the induction of *stm-l* by *pax2* [42]. The expression of *stm-1* was extremely elevated after heat shock in the injected embryo. *Pax2* mediates *stm-l* expression in medaka embryos at otolith vesicles [42]. We found high expression levels of both *pax2a* and *stm* in ovarian tissue during ovulation. This is the first finding that *pax2a* and its downstream target gene, *stm*, are co-induced in the reproductive tissue of zebrafish.

Furthermore, the biological function of all candidates underlying ovulation should be examined using recently developed gene knock-out techniques. In the current study, to demonstrate the function of the *stm* gene, we generated loss-of-function mutants using the CRISPR/Cas9 system [12, 43].

By taking advantage of the high efficiency of the CRISPR/Cas9 system, we succeeded in producing many mutant *stm* gene strains. We could find some mutants even in the F1 progeny, and produced homozygous mutants in F2 generations. The most expected *stm* mutant phenotype was the interruption of oogenesis at ovulation, and the inability to spawn eggs. However, all *stm*^-/-^ female spawn eggs, although a high percentage of unfertilized eggs and abnormal embryos were found in juveniles from *stm*^-/-^ female. We found that a percentage of F3 unfertilized eggs in the *stm*^-/-^ female × WT pair are almost the same as with the *stm*^-/-^ female × *stm*^-/-^ male pair. While, a percentage of F3 unfertilized eggs in the *stm*^-/-^ male × WT pairs are almost the same as with the WT × WT pair. Moreover, abnormal embryos from both the *stm*^-/-^ female × *stm*^-/-^ male pair and the *stm*^-/-^ female × WT pair have been detected at the blastula stage (3 HPF). An extremely higher percentage of unfertilized eggs were born from the *stm*^-/-^ female. We conclude that the unfertilized eggs are a consequence of maternal effect. The *stm* gene must be required to prepare fertilizable eggs in zebrafish after ovulation. Otolith morphogenesis has been reported to involve *stm* [40]. However, in this study, we suggest another function of *stm* gene during ovulation. The reason why only about 20–30% of eggs became unfertilized remains a mystery. Further analysis of STM proteins in ovarian tissues should be conducted.

In conclusion, this study provides eight new ovulation-related genes. Up-regulation of the eight candidates in both artificially and naturally ovulation-induced samples was confirmed. We validated the *in viv*o function of one candidate gene (*stm*) from our list. The *stm* mutants were produced using the CRISPR/Cas9 system. Unfertilized eggs and abnormal embryos at the early stage of development were produced from *stm* homozygous mutants. *Stm* appears not to play an important role in ovulation, but does support fertilization.

## Supporting information

S1 Table The list of selected genes as candidates for ovulation-inducing genes. Average relative expression levels for each gene in 4 treated groups were indicated.
(XLSX)
